# Supplementary material for: Spatial attention enhances network, cellular and subthreshold responses in mouse visual cortex
Source: Nat Commun. 2020 Jan 24;11:505. doi: 10.1038/s41467-020-14355-4 (PMC6981183; doi:10.1038/s41467-020-14355-4)
Supplement: Supplementary file 1 — Supplementary Information [file 41467_2020_14355_MOESM1_ESM.pdf]

Spatial attention enhances network, cellular and subthreshold responses in mouse visual cortex

Speed et al.

Supplementary Figures 1-11

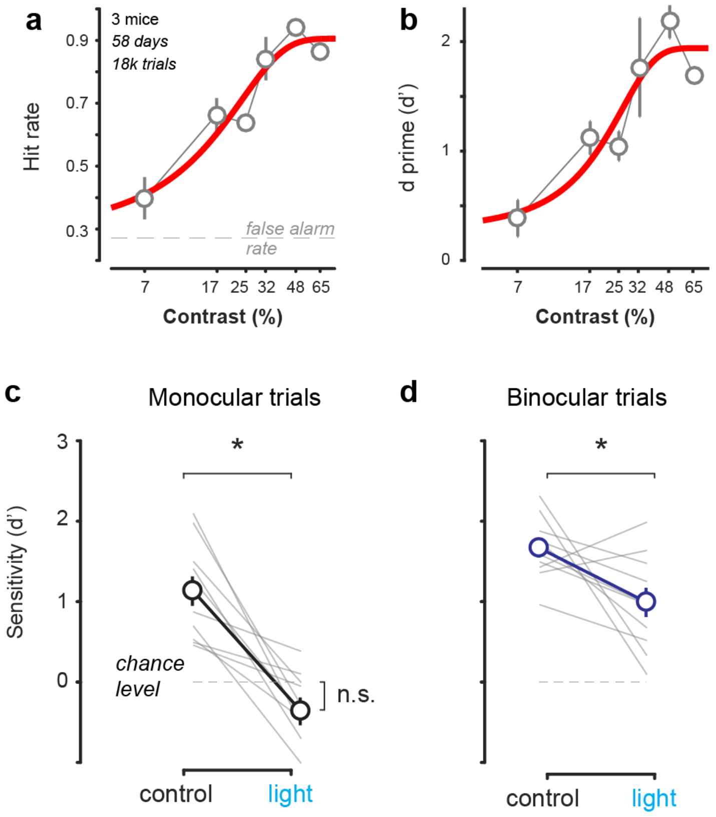

<u>Supplementary Figure 1:</u> Monocular detection performance depends on Stimulus contrast and V1 activity

- **a.** Monocular detection hit rate increased as a function of contrast, n = 3 mice, 58 behavioral sessions, 18,000 trials
- **b.** As in a, for d prime (d') as a function of contrast.
- **c.** Optogenetic inhibition of monocular V1 (interleaved on 25% of trials) significantly impaired monocular detection sensitivity (d', 1.13 ± 0.19 to -0.36 ± 0.18; mean ± SD; $p < 0.01$; Wilcoxon signed-rank test; n = 10 days and 1575 trials). During inactivation, d' was not significantly different from chance level ($p < 0.01$, sign test). Data from two PV-cre x A1-32-ChR2 mice. Visual stimulus contrast varied between 10 and 65%.
- **d.** During same behavioural sessions, interleaved blocks of binocular detection remained significantly above chance level during monocular V1 inactivation (0.99 ± 0.19, $p < 0.001$, sign test). Inactivation significantly reduced detection sensitivity versus control trials (1.67 ± 0.12, $p = 0.04$ signed rank test). Visual stimulus contrast varied between 5 and 35%.

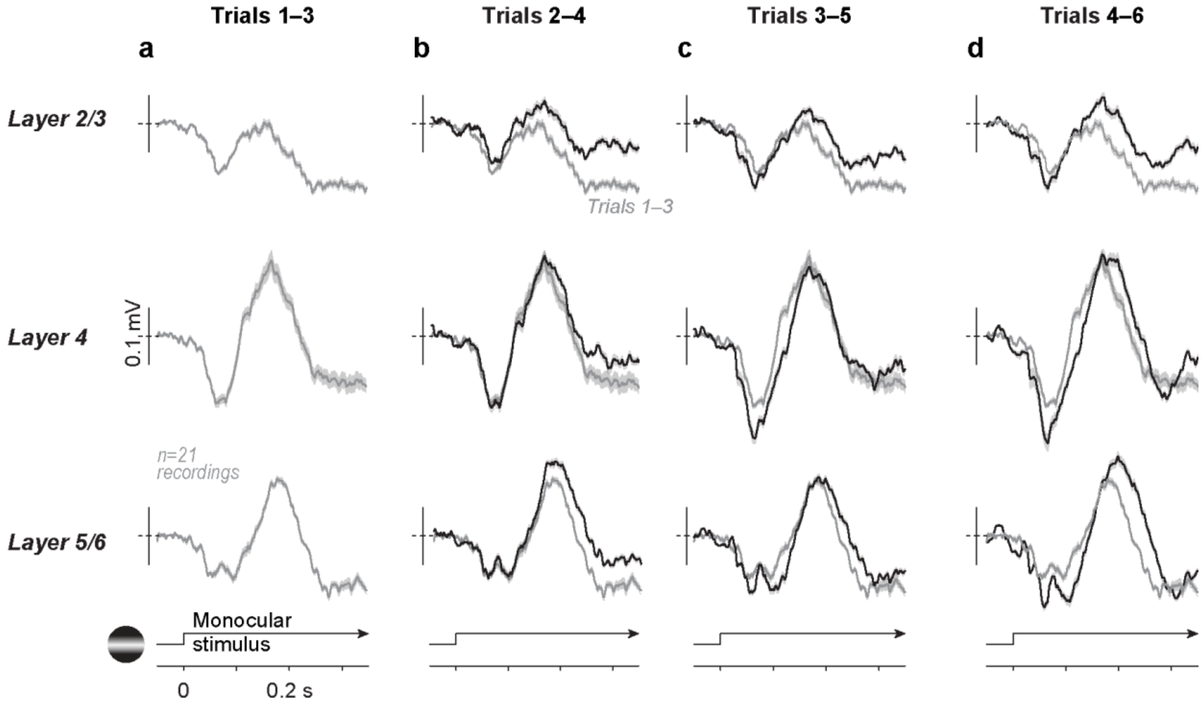

Supplementary Figure 2: Enhancement of V1 laminar LFP responses during behavioural improvements

a. Local field potential (LFP) response across layers to stimulus onset on $Trials_{(1–3)}$ during monocular detection block. Mean ± sem (15 mice, 21 recording sessions from monocular V1). Layers identified with current source density analysis[1].

b. LFP response from same recordings as a, on $Trials_{(2–4)}$ (black). $Trials_{(1–3)}$ response in grey.

c. As in a, on $Trials_{(3–5)}$ (black).

d. As in a, on $Trials_{(4–6)}$ (black).

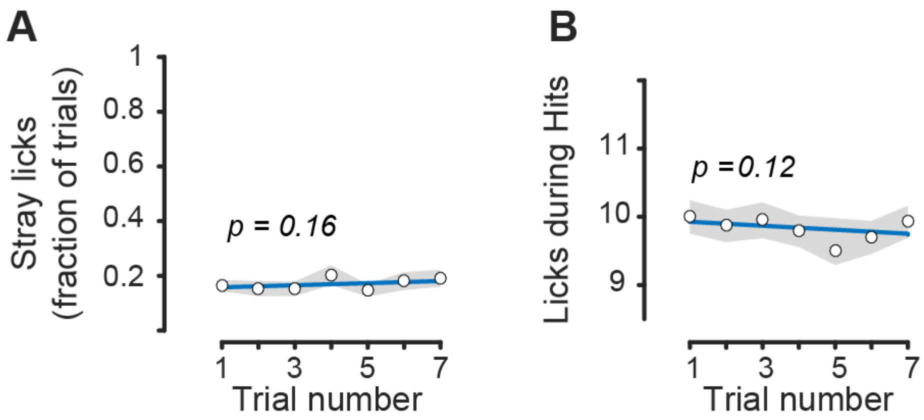

Supplementary Figure 3: Behavioral improvements are not the result of changes in motivation

a. Fraction of trials with unrewarded licks during the inter-stimulus interval as a function of trial position within the block (mean ± sem). The rate of unrewarded licks in the inter-stimulus interval does not change significantly throughout the block of trials (*p=0.16*, t-test for significant correlation)

b. Number of consummatory licks following reward delivery as a function of trial position within block (mean ± sem). The number of licks does not change significantly throughout the block of trials (*p* = *0.12*, t-test for significant correlation)

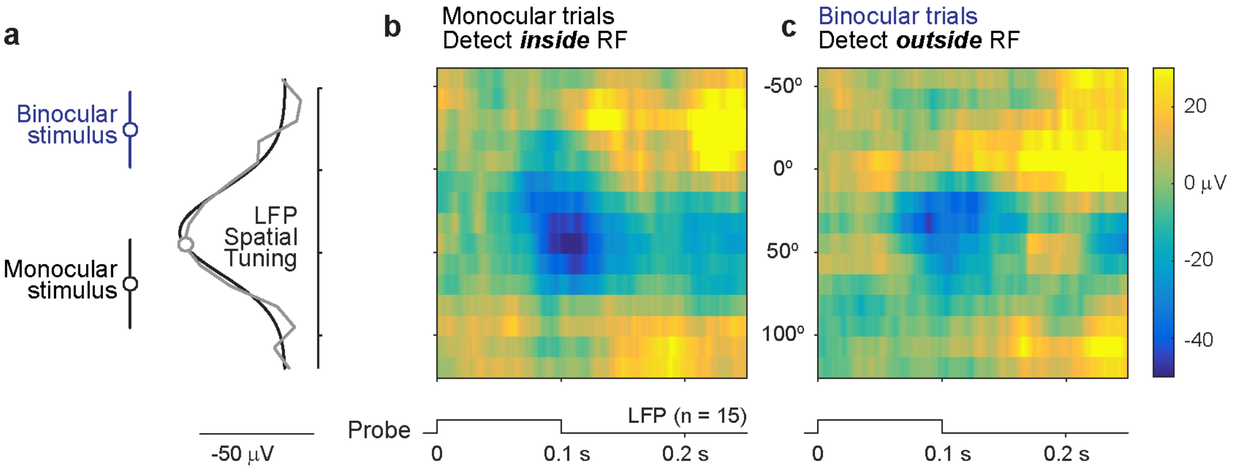

Supplementary Figure 4: Spatially specific modulation of LFP responses elicited by spatial context

a. Left, distribution of stimulus locations and extent in Binocular (blue; -24 ± 24˚; centre ± 2σ width of Gabor) and Monocular (71 ± 28˚) detection blocks across all recordings (n = 21 mice). Right, average LFP response (abscissa, grey line) to bars presented across the entire visual field (ordinate). Bars 10˚ wide, 100% contrast, 0.1s duration, presented outside of task. The location of maximum LFP sensitivity overlapped with the monocular stimulus location.

b. Space-time receptive field profiles of LFP recordings in a, aligned to probe stimuli preceding correct detection trials. On monocular detection trials, attention is elicited inside the LFP receptive field (RF).

c. Same recordings as in b, during interleaved binocular detection blocks with attention elicited outside the RF. Note that there is no response in the binocular stimulus location. LFP responses on detect inside RF trials (-20.9 ± 0.73 µV) significantly larger (more negative) than detect outside RF responses (-15.6 ± 0.53 µV). Mean ± sem averaged across Monocular RF (0 to 100˚) and time (0.075 to 0.15s; $p < 0.001$ signed rank test).

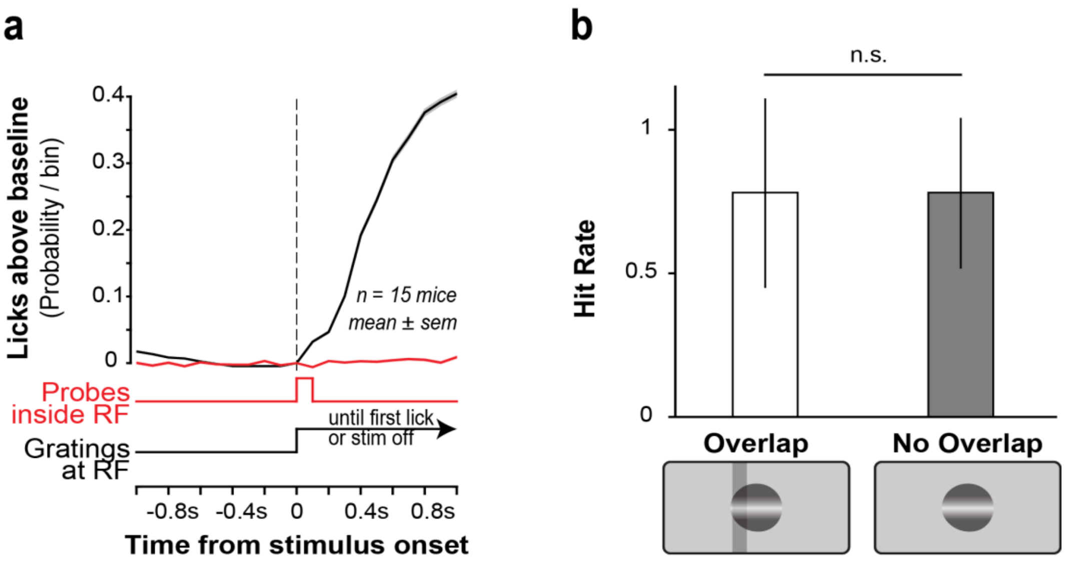

Supplementary Figure 5: Probe stimuli have no effect on behaviour.

a. Evoked lick rate in response to probe stimuli (red), vs grating stimuli (black).

b. Hit rate in response to grating stimuli on trials where probe stimuli are present overlapping the detected grating (0.78 ± 0.33; mean ± sd) vs when no overlap occurred (0.78 ± 0.26). No significant effect of overlapping probe and grating on hit rate (p=0.31 signed rank test).

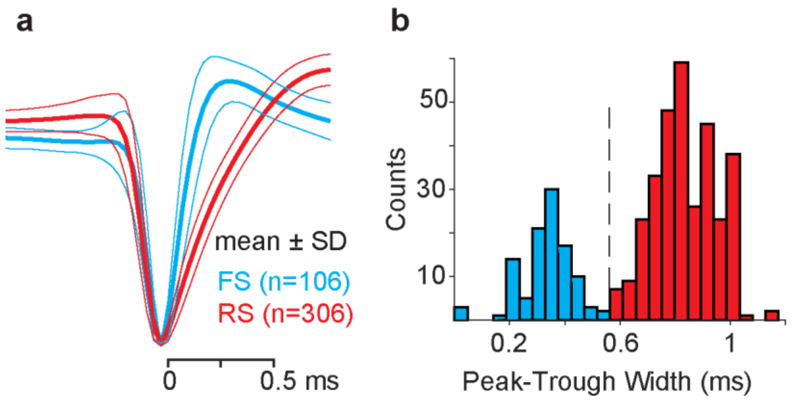

Supplementary Figure 6: Classification of RS and FS neurons

a. Average waveforms of all recorded neurons, classified as fast spiking (FS, cyan) or regular spiking (RS, red). Mean ± SD. RS Neurons: n = 306 (225 recorded during behaviour, 81 in passive awake conditions for contrast responses in Supplementary Fig. 9). FS Neurons: n = 106 (67 recorded during behaviour, 39 in passive awake conditions for contrast responses, Supplementary Fig. 9).

b. Histogram of all spike widths from a, showing clear bimodality of population. Dashed line (0.57 ms) separates the two populations.

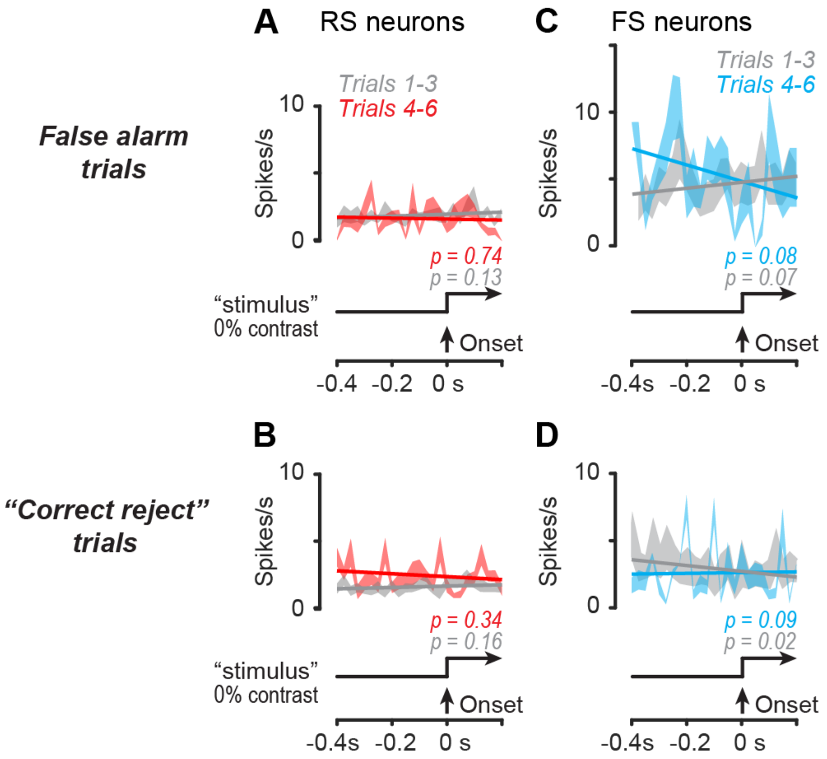

Supplementary Figure 7: Temporal modulation of Excitatory and Inhibitory activity is stimulus dependent

a. Average RS firing rate leading up to stimulus onset for 0 contrast false alarm trials early in the block (grey) compared to later in the block (red). No significant increase in firing rate preceding the stimulus.

b. Average RS firing rate leading up to stimulus onset for 0 contrast false alarm trials early in the block (grey) compared to later in the block (red). No significant increase in firing rate preceding the stimulus.

c-d. As in a-b for FS neurons

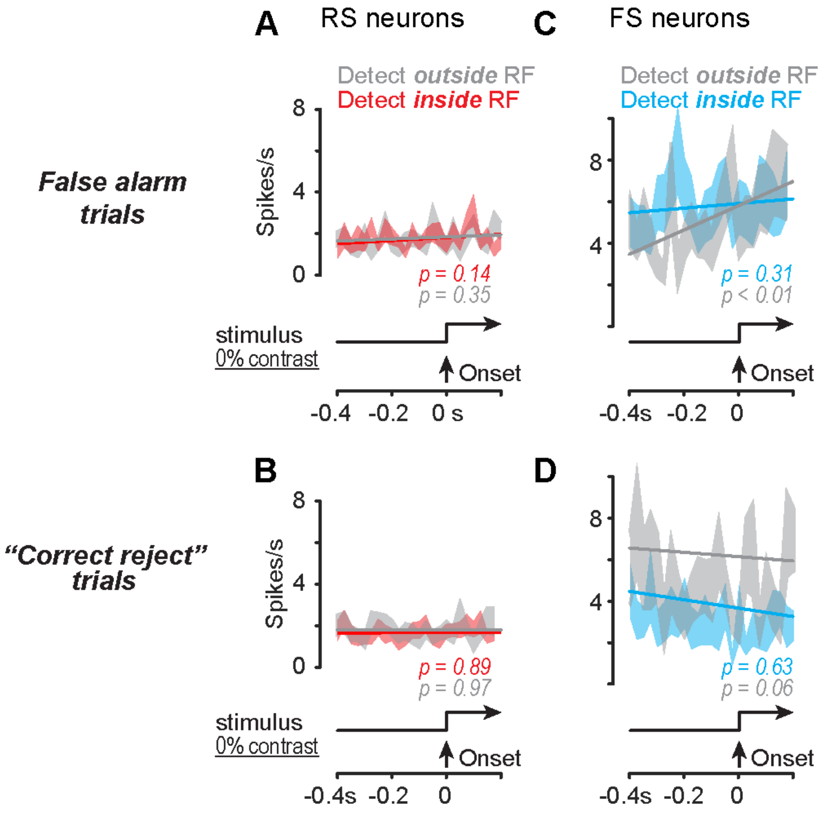

Supplementary Figure 8: Spatial modulation of excitatory and inhibitory activity is stimulus dependent

a. Average RS firing rate leading up to stimulus onset for 0 contrast false alarm trials during "detect out" blocks (grey) compared to "detect in" blocks (red). No significant increase in firing rate preceding the stimulus.

b. As in A for 0% contrast correct reject trials

c-d. As in a-b for FS neurons, Significant increase in firing rate leading up to "stimulus" presentation only observed for FS neurons in "detect out" blocks (p <0.01, t-test for correlation).

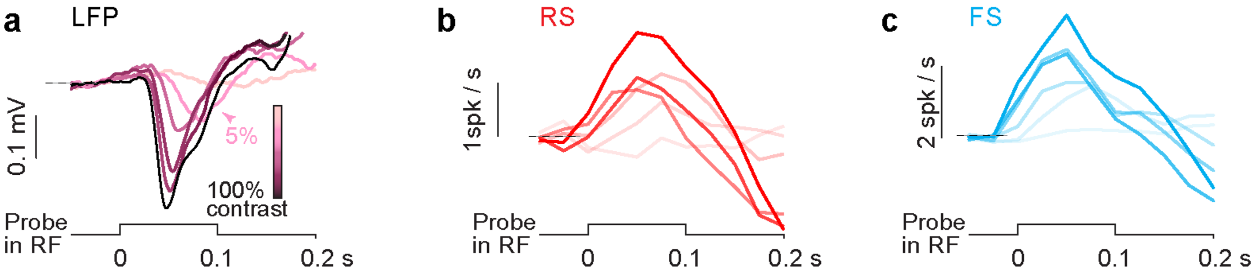

Supplementary Figure 9: Contrast response functions of LFP, RS, and FS neurons

a. LFP responses to probe stimuli of varying contrasts presented in the center of the receptive field (see Methods). Responses from L4 in awake, out of task conditions (n = 4 mice, 7 recordings). Contrast range [± 2.5, 5, 12.5, 25, 37.5, 50] for dark or bright bars from background grey. Probe stimuli presented in task at ±5% contrast.

b. As in a, for regular spiking (RS) neurons (n = 81)

c. As in a, for fast spiking (FS) neurons (n = 39).

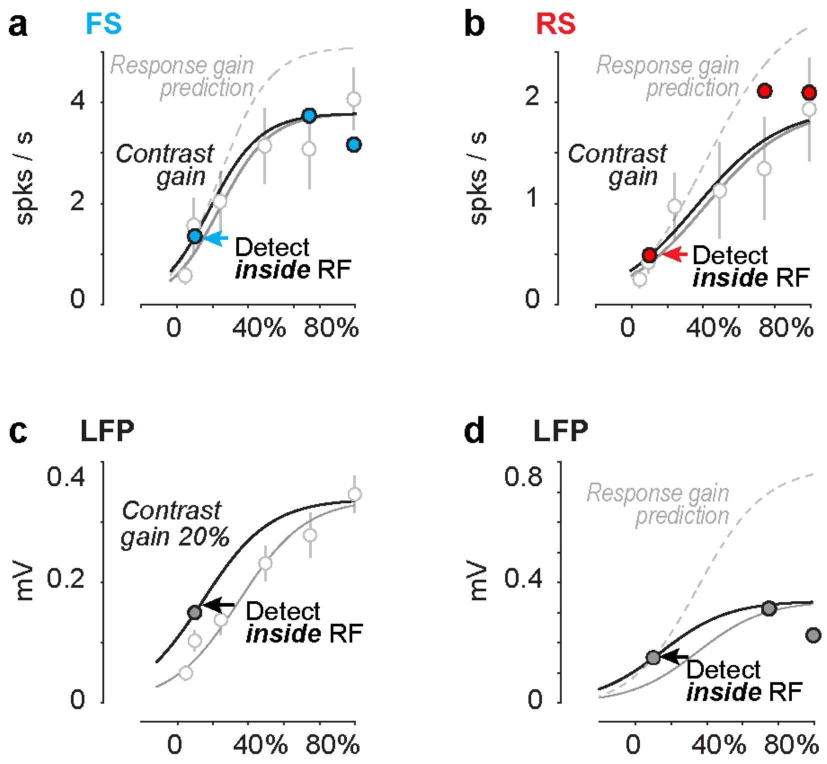

Supplementary Figure 10: Spatially selective response enhancement resembles contrast gain

a. FS neuron contrast response function (CRF) to probes (grey circles, n = 39 outside of task; Supplementary Fig. 9). Contextual enhancement of probe responses (cyan arrow) equivalent to +5.4% contrast gain (black curve). Contrast gain model accurately predicts contextual modulation at high contrast (cyan circles; 1.6 ± 2.0% and 15.9 ± 1.0% error, mean ± sem), versus multiplicative response gain model (dashed grey; 23.1 ± 2.0% and 36.3 ± 1.0% error; [predicted-measured] / measured, per trial). High contrast contextual modulation measured for gratings during task (75%, 100% contrast).

b. Same as a, for RS neurons (n=81). Contextual enhancement equivalent to +5.8% contrast gain (black). Contrast gain predicts high contrast responses less accurately (33.6 ± 11.7% and 16.2 ± 1.0% error) than multiplicative response gain (9.6 ± 1.4% and 4.8 ± 0.1% error).

c. Same for L4 LFP (CRF from 7 recordings, 4 mice out of task). Contextual enhancement equivalent to +20.0% contrast gain ($p < 0.05$ rank sum test; detect inside RF mean response, 0.15 mV, 95% CI for CRF [0.03 – 0.12 mV])

d. Contrast gain predicts contextual modulation of high contrast L4 LFP responses more accurately (3.4 ± 27.3% and 2.9 ± 18.2% error) than multiplicative response gain model (51.0 ± 13.0% and 54.0 ± 8.6% error).

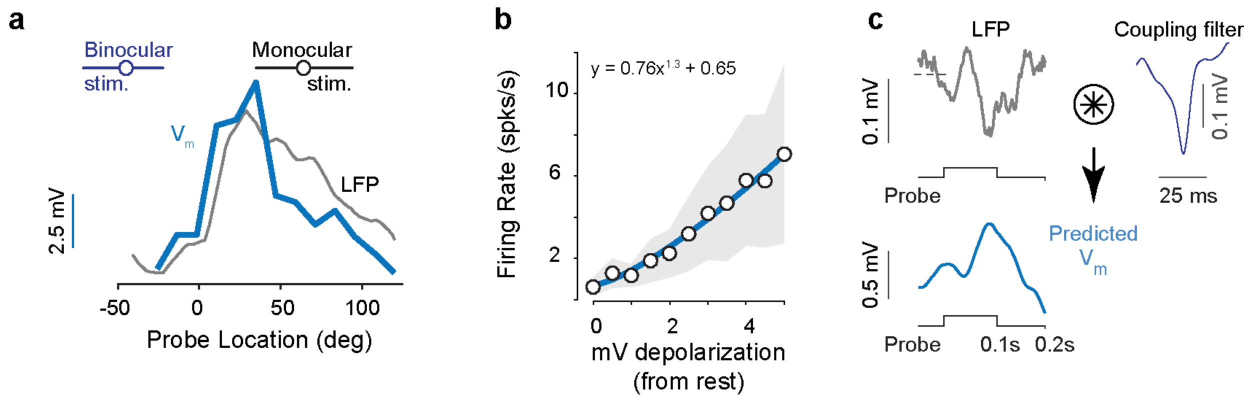

Supplementary Figure 11: Membrane potential variability, spatial tuning, and prediction from LFP

a. Spatial tuning of membrane potential (V_m, blue), calculated as maximum response to probe stimuli presented across all spatial locations during correct detection trials. LFP spatial tuning (separate experiments, same craniotomies) in grey. Both LFP and V_m spatial tuning overlaps location of monocular stimuli.

b. Calculation of V_m to spike rate transformation (power law exponent = 1.3), for neurons with stimulus evoked firing rates > 0.1 spikes/s (n = 6 neurons from awake V1 dataset in Haider et al., 2016).

c. Example single-trial prediction of V_m response (bottom) from LFP response to probe stimulus during detect inside RF trials (top). LFP trace was convolved with average coupling filter inferred from simultaneous awake LFP-V_m recordings of probe responses[2].

Supplementary References:

1. Speed, A., Del Rosario, J., Burgess, C. P. & Haider, B. Cortical State Fluctuations across Layers of V1 during Visual Spatial Perception. *Cell Rep* **26**, 2868-2874 e2863, doi:10.1016/j.celrep.2019.02.045 (2019).
2. Haider, B., Schulz, D. P., Hausser, M. & Carandini, M. Millisecond Coupling of Local Field Potentials to Synaptic Currents in the Awake Visual Cortex. *Neuron* **90**, 35-42, doi:10.1016/j.neuron.2016.02.034 (2016).
