# Supplementary material for: Spatial attention enhances network, cellular and subthreshold responses in mouse visual cortex
Source: Nat Commun. 2020 Jan 24;11:505. doi: 10.1038/s41467-020-14355-4 (PMC6981183; doi:10.1038/s41467-020-14355-4)
Supplement: Supplementary file 3 — Reporting Summary [file 41467_2020_14355_MOESM3_ESM.pdf]

nature research

Corresponding author(s): Haider, Bilal

Last updated by author(s): Dec 19, 2019

Reporting Summary

Nature Research wishes to improve the reproducibility of the work that we publish. This form provides structure for consistency and transparency in reporting. For further information on Nature Research policies, see Authors & Referees and the Editorial Policy Checklist .

Statistics

For all statistical analyses, confirm that the following items are present in the figure legend, table legend, main text, or Methods section.

n/a	Confirmed	
☐	☒	The exact sample size (*n*) for each experimental group/condition, given as a discrete number and unit of measurement
☐	☒	A statement on whether measurements were taken from distinct samples or whether the same sample was measured repeatedly
☐	☒	The statistical test(s) used AND whether they are one- or two-sided<br>*Only common tests should be described solely by name; describe more complex techniques in the Methods section.*
☐	☒	A description of all covariates tested
☐	☒	A description of any assumptions or corrections, such as tests of normality and adjustment for multiple comparisons
☐	☒	A full description of the statistical parameters including central tendency (e.g. means) or other basic estimates (e.g. regression coefficient) AND variation (e.g. standard deviation) or associated estimates of uncertainty (e.g. confidence intervals)
☐	☒	For null hypothesis testing, the test statistic (e.g. *F*, *t*, *r*) with confidence intervals, effect sizes, degrees of freedom and *P* value noted<br>*Give P values as exact values whenever suitable.*
☒	☐	For Bayesian analysis, information on the choice of priors and Markov chain Monte Carlo settings
☒	☐	For hierarchical and complex designs, identification of the appropriate level for tests and full reporting of outcomes
☒	☐	Estimates of effect sizes (e.g. Cohen's *d*, Pearson's *r*), indicating how they were calculated

Our web collection on statistics for biologists contains articles on many of the points above.

Software and code

Policy information about availability of computer code

Data collection	MATLAB 2015b; Blackrock Microsystems Cereplex Direct
Data analysis	MATLAB 2015b, 2016; KlustaSuite (Rossant et al., 2016)

For manuscripts utilizing custom algorithms or software that are central to the research but not yet described in published literature, software must be made available to editors/reviewers. We strongly encourage code deposition in a community repository (e.g. GitHub). See the Nature Research guidelines for submitting code & software for further information.

Data

Policy information about availability of data

All manuscripts must include a data availability statement. This statement should provide the following information, where applicable:
- Accession codes, unique identifiers, or web links for publicly available datasets
- A list of figures that have associated raw data
- A description of any restrictions on data availability

All data structures and code necessary to reproduce the results of the current study are available from the corresponding author upon reasonable request.

Field-specific reporting

Please select the one below that is the best fit for your research. If you are not sure, read the appropriate sections before making your selection.

☒ Life sciences ☐ Behavioural & social sciences ☐ Ecological, evolutionary & environmental sciences

For a reference copy of the document with all sections, see nature.com/documents/nr-reporting-summary-flat.pdf

Life sciences study design

All studies must disclose on these points even when the disclosure is negative.

Sample size	Sample sizes were not pre-calculated, but are comparable to similar studies in the field.
Data exclusions	During spike sorting, poorly isolated waveforms (multi-unit clusters) were excluded from further analyses. All mice trained on multiple position spatial detection learned the task, and all recording sessions during behavior were analyzed.
Replication	Multiple instances of replication were successful. Main findings (both behavioral and neural) were replicated in separate cohorts of mice trained months apart by different individuals, and were replicated across multiple experimental set-ups.
Randomization	Randomization of experimental subjects was not necessary for experimental design (within subjects analysis).
Blinding	Blinding was not necessary for the experimental design.

Reporting for specific materials, systems and methods

We require information from authors about some types of materials, experimental systems and methods used in many studies. Here, indicate whether each material, system or method listed is relevant to your study. If you are not sure if a list item applies to your research, read the appropriate section before selecting a response.

Materials & experimental systems

n/a	Involved in the study	
☒	☐	Antibodies
☒	☐	Eukaryotic cell lines
☒	☐	Palaeontology
☐	☒	Animals and other organisms
☒	☐	Human research participants
☒	☐	Clinical data

Methods

n/a	Involved in the study	
☒	☐	ChIP-seq
☒	☐	Flow cytometry
☒	☐	MRI-based neuroimaging

Animals and other organisms

Policy information about studies involving animals; ARRIVE guidelines recommended for reporting animal research

Laboratory animals	All experiments used male mice (4-6 weeks old; reverse light cycle individual housing; bred in house), either of C57BL6J strain (RRID: IMSR_JAX:000664), or offspring of PV-cre mice (RRID: IMSR_JAX:017320) crossed with Ai-32-ChR2 mice (RRID: IMSR_JAX:024109).
Wild animals	N/A
Field-collected samples	N/A
Ethics oversight	All procedures were approved by the Institutional Animal Care and Use Committee at the Georgia Institute of Technology and were in agreement with guidelines established by the National Institutes of Health and the Animals (Scientific Procedures) Act 1986 (UK).

Note that full information on the approval of the study protocol must also be provided in the manuscript.
